# Improving the isolated microspore culture in eggplant (*Solanum melongena* L.) with amino acid nutrition

**DOI:** 10.1371/journal.pone.0286809

**Published:** 2023-06-08

**Authors:** Mozhgan Hashemi, Ahmad Moieni, Mohammad Sadegh Sabet

**Affiliations:** Faculty of Agriculture, Department of Plant Genetics and Breeding, Tarbiat Modares University, Tehran, Iran; United States Department of Agriculture, UNITED STATES

## Abstract

It has been proposed that the composition of the culture medium, especially its amino acids, is an important part of getting microspore androgenesis to occur in some plants. However, there have been far fewer studies done on the Solanaceae family. In this study, we studied what happened to eggplant microspore culture when we mixed casein hydrolysate (0 and 100 mg L^-1^) with four amino acids: proline (0, 100, 500, and 900 mg L^-1^), glutamine (0 and 800 mg L^-1^), serine (0 and 100 mg L^-1^), and alanine (0 and 100 mg L^-1^). The results showed that a combination of 800 mg L^-1^ of glutamine, 100 mg L^-1^ of serine, 100 mg L^-1^ of casein hydrolysate, and 500 mg L^-1^ of proline produced the maximum number of calli per Petri dish (938). Calli had a globular shape and a compact appearance when formed in media containing 500 mg L^-1^ of proline (alone or combined with serine, alanine, and/or casein hydrolysate). Most of these structures were observed in a medium with 500 mg L^-1^ of proline, 100 mg L^-1^ of casein hydrolysate, and 100 mg L^-1^ of serine. We also investigated what happened when gum arabic (2400, 2600, 3600, 4600, and 5600 mg L^-1^) was combined with proline (0 and 500 mg L^-1^), casein hydrolysate (0 and 100 mg L^-1^), and glutamine (0, 400, and 800 mg L^-1^). The findings demonstrated the involvement of proline in the increase of calli. Overall, the results give us new information about how amino acids work in eggplant microspore culture and suggest that proline can move this plant’s microspore androgenesis pathway forward.

## 1. Introduction

In programs to produce hybrid seeds, the first step is to make sure there are inbred lines. The doubled haploid method is better than the classical ways of making inbred lines because it saves time and money and gives access to 100% homozygosity. *In vitro* androgenesis is the most practical method for producing doubled haploid plants [1]. This method, which includes anther culture and isolated microspore culture, is one of the most common ways to create doubled haploid plants from eggplant [2]. Isolated microspore culture is better because it has many benefits, such as the ability to remove the walls of the anther, access to the nutrients that microspores need to grow, and the ability to study the development and growth of microspore embryos [3].

Gu [4] reported the first eggplant microspore culture. The next findings were reported by Miyoshi [5]. He made a microspore culture protocol that included starvation and heat stress of 35°C for three days. The microspores were then cultured in Nitsch and Nitsch (NLN) medium with 2% sucrose, 0.5 mg L^-1^ naphthalene acetic acid (NAA), and 0.5 mg L^-1^ benzyl adenine (BA). Bal *et al*. [6] promoted the microspore culture of eggplant using a different method. Their instruction was based on the tobacco microspore culture protocol. Their results were limited to observing symmetric cell divisions and producing multicellular structures.

Corral Martinez and Seguıí-Simarro used Miyoshi’s protocol again in 2012 [7]. Because of this new research, globular embryos formed, but they turned into a callus in the end. Then, Corral Martinez and Seguıí-Simarro [8] reported that using substances like polyethylene glycol, epibrassinolide, and gum arabic can help improve microspore embryogenesis and calli proliferation. Rivas-Sendra *et al*. [9] showed that the intensity of light that hits donor plants affects the viability of eggplant microspores. In another study, the levels of endogenous plant growth regulators (cytokinins, auxins, gibberellins, jasmonic acid, abscisic acid, and salicylic acid) were investigated in eggplant microspore embryogenesis. Different accumulation patterns of plant growth regulators during microspore culture were linked to different embryogenic responses [10]. Even though these studies were done, the problems with the eggplant microspore embryogenesis pathway (globular embryos not changing into later embryonic stages) still exist. Because of this, more should be done to find substances that could improve microspore embryogenesis induction. The composition of the culture medium, especially with macromolecules like nitrogen sources, has helped a lot to induce haploidy. Nitrogen can be added to the culture medium in two forms: organic (amino acids) and inorganic (NO_3_^-^ or NH_4_^+^) [11]. Proline accumulates in plants under both stressed and non-stressed conditions. In wheat [12] has been shown that this amino acid effectively induces androgenesis. Many *in vitro* cells that divide quickly can get energy from glutamine, which is a readily available amino acid [13]. Also, Dahrendorf *et al*. [14] showed that Norwegian poplar embryogenic cultures proliferated the fastest and produced the most mature embryos in a medium with glutamine but no mineral nitrogen. Serine (Ser) plays an essential role in metabolism and signaling in living organisms [15]. It has been reported that serine and proline can stimulate embryo formation during androgenesis [16]. Alanine is a glucogenic amino acid used a lot in protein biosynthesis, and it is made from branched amino acids like leucine, isoleucine, and valine [17].

Casein hydrolysate contains phosphate, calcium, vitamins, microelements, and, most importantly, 18 amino acids. It can improve plant growth processes [18]. Gum arabic is a complex polysaccharide that is branched, neutral, or slightly acidic and has a mix of potassium, calcium, and magnesium salts. Acacia senegal gum arabic contains roughly 44% galactose, 27% arabinose, 13% rhamnose, 16% glucuronic acid, and 4-O-methyl glucuronic acid. It also contains 2–3% peptide as an integral part of its structure [19,20]. Borderies *et al*. [21] reported the secretion of Arabinogalactan proteins (AGPs) into the medium during maize microspore culture. They suggested that AGPs may stimulate microspores embryogenesis from non-responsive genotypes. Corral-Martı´nez and Seguı´-Simarro [8] also showed that AGPs could be used for eggplant microspore callogenesis.

This study looked at how casein hydrolysate, gum arabic, and four amino acids (proline, glutamine, serine, and alanine) affected a culture of isolated eggplant microspores.

## 2. Material and methods

### 2.1 Plant material and plant growth conditions

An eggplant F1 hybrid cultivar, Ricarda, was used in this experiment. The seeds were grown for 1.5 months in a controlled plant growth chamber at 25°C with a 16-hour photoperiod in nursery trays with peat moss and perlite (3:1, v/v). The plants were then moved to a plastic greenhouse (GPS coordinates: 51°09 E longitude and 35°44 N altitude, 1265 m above sea level), where the daytime temperature was 22°C, and the nighttime temperature was 19°C. The plants were exposed to natural light during the day. Plants were fertilized once every three weeks with a complete fertilizer (20N-20P-20K) and irrigated every three days.

### 2.2 Collection of donor buds, isolation, and culture of microspores

From late June to late September, flower buds were picked when the microspores were in the appropriate stages of growth (late uninucleate and early binucleate) and washed for 5 min with 3–4 drops of liquid dish soap. Then, they were put in a laminar air flow hood and surface-sterilized in 70% ethanol for 5 min, followed by 20 min in 1% (w/v) bleach with two drops of Tween 20 (Merck, Darmstadt, Germany), and then rinsed three times with sterile distilled water. Buds’ anthers were excised and blended in 20 mL of pre-cooled sterile deionized water using a Waring Blender (MC2-37 to 110 mL, Clarkson Laboratory and Supply Inc., Chula Vista, CA), twice at 10 s and 5 s on medium speed, respectively. The blender cup had already been chilled in the fridge. The suspended extract was filtered through a 40 μm nylon mesh sieve and centrifuged at 113 g for 4 min. After that, it was washed three times with sterile, deionized water that had been chilled. The isolated microspores were suspended in sterile, deionized water at a density of about 200,000 per ml, spread out in 6-cm plastic Petri dishes, and incubated at 35°C in the dark for three days. After being put through starvation and heat, the microspores were collected by centrifuging them at 113 g for 3 min. Then, they were resuspended in a liquid NLN culture medium and incubated in the dark at 25°C for a month.

### 2.3 Study of the effects of casein hydrolysate and the amino acids glutamine, serine, alanine, and proline

This experiment was done to study how different concentrations of glutamine (0 and 800 mg L^-1^), serine (0 and 100 mg L^-1^), alanine (0 and 100 mg L^-1^), proline (0, 100, 500, and 900 mg L^-1^), and casein hydrolysate (0 and 100 mg L^-1^) affected the induction of androgenic structures in eggplant microspore culture. These substances were added to the NLN medium (pH 5.9) after the combined stress of starvation and heat. The media without the vitamins were sterilized by autoclave for 20 min at 121°C. Then, the filter-sterilized vitamins were added to the autoclaved media, which was cooled to 60°C.

### 2.4 Study of the effects of gum arabic, glutamine, proline, and casein hydrolysate

This experiment was conducted after the first experiment to know how the different amounts of gum arabic (0, 2400, 2600, 3600, 4600, and 5600 mg L^-1^), glutamine (0, 400, and 800 mg L^-1^), proline (0 and 500 mg L^-1^), and casein hydrolysate (0 and 100 mg L^-1^) affected the induction of microspore androgenesis. The rest conditions were identical to those used in the previous experiment.

### 2.5 Data collection and statistical analysis

In a factorial experiment with a completely randomized design, the experiments were done with five replications of each treatment under the same conditions and simultaneously. After inducing androgenesis, the microspores made calli or globular embryos that later became calli. So, after one month of microspore culture, three characteristics were measured: the total number of calli, the number of calli 1–2 mm in diameter, and the number of calli > 2 mm per Petri dish. The data were analyzed using the Wald Chi-Squared test in non-parametric generalized linear models. Pairwise comparisons were conducted using the Kruskal-Wallis test. These methods allowed us to extract meaningful insights from the data without making any assumptions about the underlying distribution or structure of the dataset. Through this comprehensive approach, we were able to identify significant patterns and trends, which helped us draw robust conclusions and make informed decisions. These tests were done in SPSS Statistics version 16.0 and SAS version 9.0. All figures for the mean comparisons were made by GraphPad Prism 7 software.

## 3. Results

### 3.1 Effects of casein hydrolysate, glutamine, alanine, serine, and proline on eggplant microspore response

In this experiment, the effects of different concentrations of casein hydrolysate and four amino acids, including glutamine, alanine, serine, and proline, on inducing androgenesis in eggplant microspore culture were studied. The results of the Wald χ^2^-test (Table 1) showed that all five-way interaction effects on the total number of calli, the number of calli 1–2 mm, and the number of calli > 2 mm traits were highly significant (*p* < 0.01). Therefore, mean comparisons were carried out only for these interaction effects.

**Table 1:** Maximum likelihood analysis of variance for the influences of different experimental factors on the microspore androgenesis of *Solanum melongena* L.

Source of Variation	d.f.	χ2		
		Total number of calli / Petri dish	Number of calli 1–2 mm/ Petri dish	Number of calli > 2 mm/ Petri dish
Glu	1	13094.4**	84.0**	0.0^ns^
Ala	1	356778.8**	7860.6**	0.2^ns^
Ser	1	90484.8**	316.0**	0.8^ns^
Ch	1	413640.7**	31126.0**	9.1**
Pro	3	2394022.3**	142642.9**	344.9**
Glu × Ala	1	175.5**	12.0**	12.8**
Glu × Ser	1	10683.7**	189.1**	120.0**
Glu × Ch	1	482.6**	42.0**	0.1^ns^
Glu × Pro	3	244752.2**	25839.2**	305.6**
Ala × Ser	1	90821.5**	5780**	31.2**
Ala × Ch	1	101638.1**	2633.5**	23.1**
Ala × Pro	3	1000864.3**	26887.7**	59.4**
Ser × Ch	1	81249.3**	99.0**	37.8**
Ser × Pro	3	646347.7**	17951.7**	188.6**
Ch × Pro	3	863971.3**	49166.5**	13.5**
Glu × Ala × Ser	1	4658.8**	101.2**	36.4**
Glu × Ala × Ch	1	10317.1**	1575.3**	127.5**
Glu × Ala × Pro	3	10245.4**	1959.0**	40.3**
Glu × Ser × Ch	1	46104.0**	6319.0**	25.3**
Glu × Ser × Pro	3	42698.0**	3427.1**	220.1**
Glu × Ch × Pro	3	67011.3**	6092.5**	0.1^ns^
Ala × Ser × Ch	1	169510.0**	12903.2**	12.0**
Ala × Ser × Pro	3	455522.5**	32513.6**	151.9**
Ala × Ch × Pro	3	362334.4**	10073.8**	282.7**
Ser × Ch × Pro	3	364973.5**	3201.4**	79.1**
Glu × Ala × Ser × Ch	1	81888.0**	3591.2**	30.0**
Glu × Ala × Ser × Pro	3	60126.3**	6500.2**	228.3**
Glu × Ala × Ch × Pro	3	13083.9**	1058.6**	135.7**
Glu × Ser × Ch × Pro	3	260600.5**	34691.4**	46.4**
Ala × Ser × Ch × Pro	3	354583.6**	22281.0**	70.9**
Glu × Ala × Ser × Ch × Pro	3	390350.0**	18016.0**	180.5**

Glu, Glutamine; Ala, Alanine; Ser, Serine; Ch, Casein hydrolysate; Pro, Proline.

**P < 0.01.

^ns^ *P* > 0.05.

**Fig 1:**
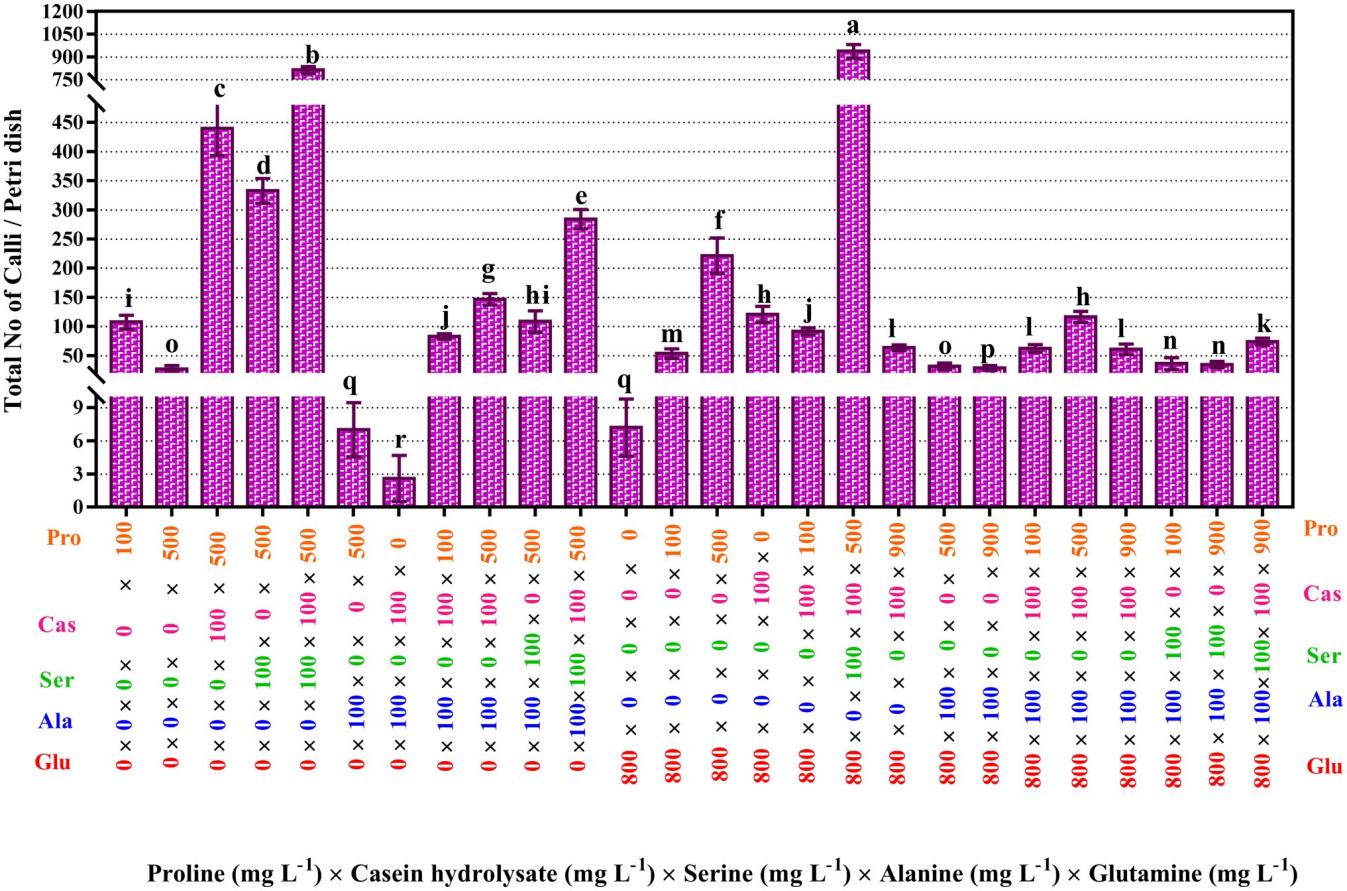
Interaction effects of proline, casein hydrolysate, serine, alanine and glutamine on a total number of calli. The studied concentrations include proline (0, 100, 500, and 900 mg L^-1^) × casein hydrolysate (0 and 100 mg L^-1^) × alanine (0 and 100 mg L^-1^) × serine (0 and 100 mg L^-1^) × glutamine (0 and 800 mg L^-1^). According to the Kruskal-Wallis test, different letters indicate statistically significant differences. The treatment combinations that did not produce callus are not shown in the figure.

Results showed that the total number of calli was highest (938) for the 800 mg L^-1^ glutamine × 0 mg L^-1^ alanine × 100 mg L^-1^ serine × 100 mg L^-1^ casein hydrolysate × 500 mg L^-1^ proline interaction effect (Fig 1) and followed by the interaction effect of 0 mg L^-1^ glutamine × 0 mg L^-1^ alanine × 100 mg L^-1^ serine × 100 mg L^-1^ casein hydrolysate × 500 mg L^-1^ proline (814.4).

**Fig 2:**
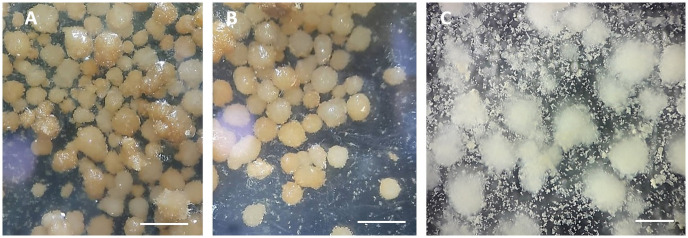
The calli produced through isolated microspore cultures in eggplant. (A and B): Calli produced in the media containing 500 mg L^-1^ of proline without glutamine. (C): Calli produced in the media containing glutamine. *Scale bars* indicate 500 μm in A and B and 700 μm in C.

Even though the highest number of calli (938) were produced with a combination of 500 mg L^-1^ of proline, 800 mg L^-1^ of glutamine, 100 mg L^-1^ of serine, and 100 mg L^-1^ of casein hydrolysate, the calli that formed in the media with 500 mg L^-1^ of proline but no glutamine had a globular shape and a compact appearance (Fig 2).

**Fig 3:**
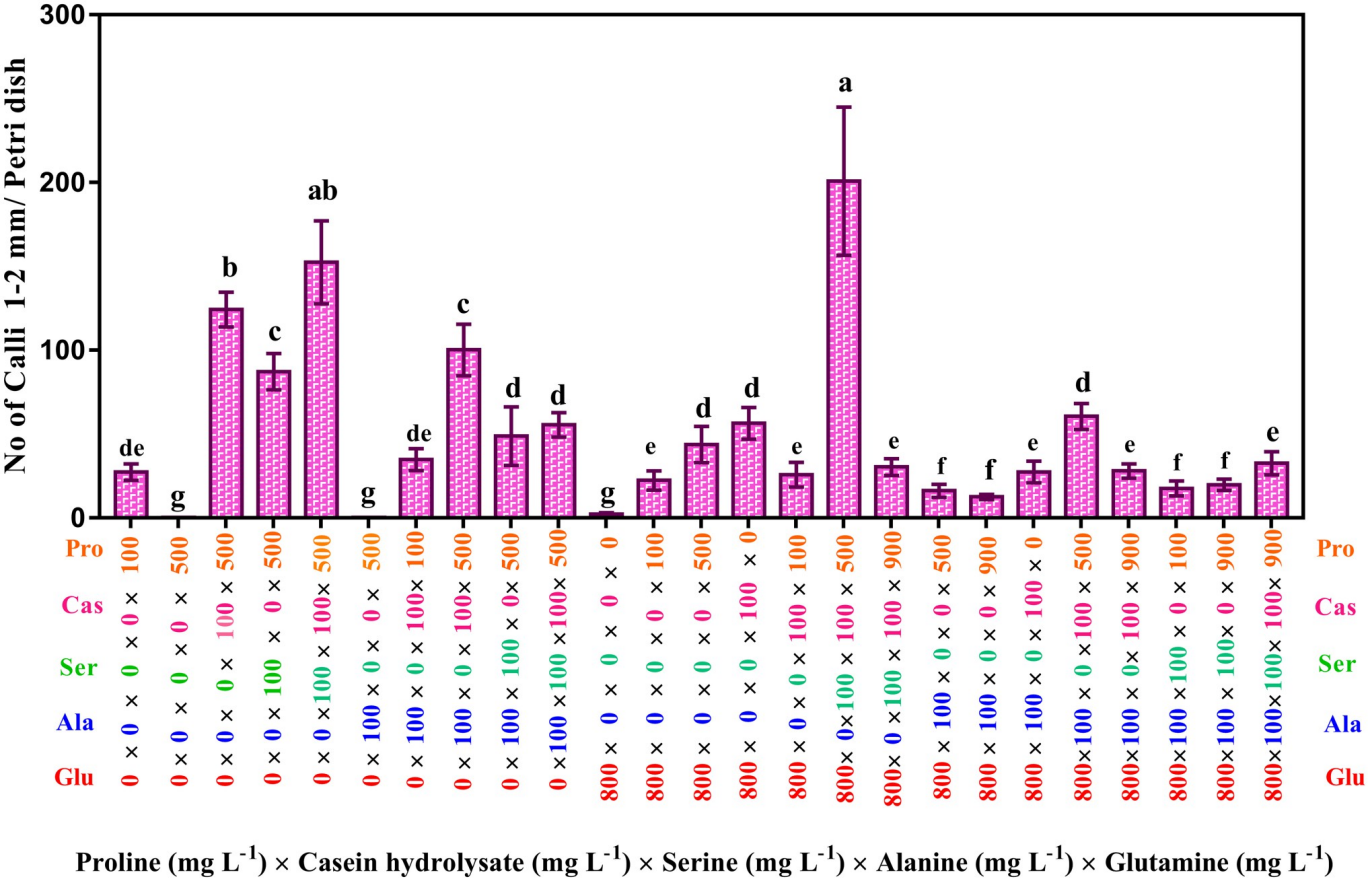
Interaction effects of proline, casein hydrolysate, serine, alanine and glutamine on producing the calli 1–2 mm. The studied concentrations include proline (0, 100, 500, and 900 mg L^-1^), casein hydrolysate (0 and 100 mg L^-1^), serine (0 and 100 mg L^-1^), alanine (0 and 100 mg L^-1^) and glutamine (0 and 800 mg L^-1^). According to the Kruskal-Wallis test, different letters indicate a statistically significant difference.

The results (Fig 3) also indicated that the medium with 800 mg L^-1^ of glutamine, 500 mg L^-1^ of proline, 100 mg L^-1^ of serine, and 100 mg L^-1^ of casein hydrolysate significantly produced the highest number of the calli 1–2 mm (200.8), followed by the medium with 500 mg L^-1^ of proline, 100 mg L^-1^ of serine, and 100 mg L^-1^ of casein hydrolysate (152.4).

**Fig 4:**
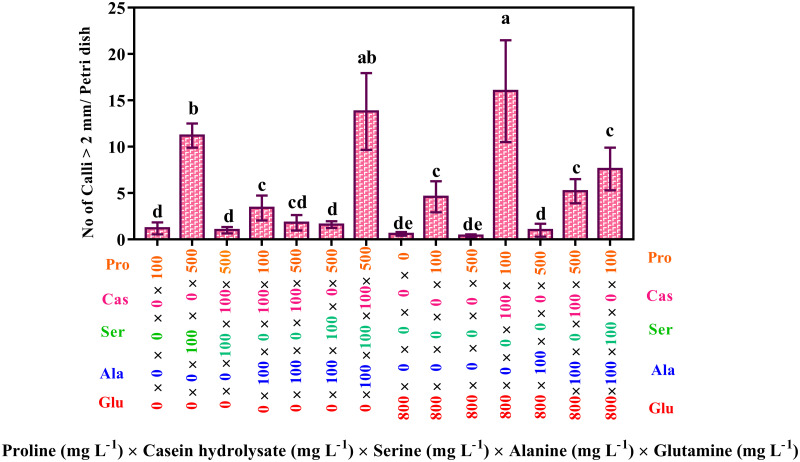
Interaction effects of proline, casein hydrolysate, serine, alanine and glutamine on producing the calli > 2mm. The studied concentrations include proline (0, 100, 500, and 900 mg L^-1^), casein hydrolysate (0 and 100 mg L^-1^), serine (0 and 100 mg L^-1^), alanine (0 and 100 mg L^-1^) and glutamine (0 and 800 mg L^-1^). According to the Kruskal-Wallis test, different letters indicate statistically significant differences.

The results (Fig 4) also showed that the medium with 800 mg L^-1^ of glutamine, 100 mg L^-1^ of proline, and 100 mg L^-1^ of casein hydrolysate produced the highest number of calli > 2 mm (16), followed by the medium with 500 mg L^-1^ of proline, 100 mg L^-1^ of serine, 100 mg L^-1^ of alanine, and 100 mg L^-1^ of casein hydrolysate (13.8).

Overall, the results showed that casein hydrolysate and the three amino acids proline, serine, and glutamine had a significant role in the response to androgenesis of eggplant microspores. Combining 100 mg L^-1^ casein hydrolysate, 500 mg L^-1^ proline, 100 mg L^-1^ serine, and 800 mg L^-1^ glutamine was the best treatment for increasing microspore-derived calli; approximately 21% of these structures were between 1 and 2mm. On the other hand, by removing glutamine from this treatment, the production of calli decreased by about 13%, but most had a globular shape and a compact appearance.

### 3.2 Effects of gum arabic, casein hydrolysate, proline, and glutamine on eggplant microspore androgenesis

This experiment studied the effects of different concentrations of glutamine, casein hydrolysate, proline, and gum arabic on eggplant microspore culture. The results of the Wald χ^2^-test (Table 2) showed that the main effects of studied factors and all their interactions on the total number of calli, the number of calli 1–2 mm, and the number of calli > 2 mm were highly significant (*p* < 0.01). Therefore, mean comparisons were carried out only for the four-way interaction effects.

**Table 2:** Maximum likelihood analysis of variance for the influences of different experimental factors on the microspore androgenesis of *Solanum melongena* L.

Source of Variation	d.f.	χ2	
		Total number of calli / Petri dish	Number of calli 1–2 mm/ Petri dish
Pro	1	1016228.1**	25233.8**
Ch	1	65259.4**	9901.5**
Glu	2	58721.0**	365.4**
GA	5	2576291.9**	89424.1**
Pro × Ch	1	69250.1**	10027.7**
Pro × Glu	2	48801.5**	280.7**
Pro × GA	5	2589909.0**	90688.7**
Glu × Ch	2	251920.7**	781.0**
GA × Ch	5	1191481.7**	51427.8**
Glu × GA	10	295070.6**	1634.4**
Pro × Ch × Glu	2	264040.6**	759.5**
Pro × Ch × GA	5	1193890.9**	51382.2**
Pro × Glu × GA	10	324509.8**	2125.3**
CH × Glu × GA	10	377970.5**	1733.9**
Pro × CH × Glu × GA	10	395073.3**	1942.5**

Pro, Proline; Ch, Casein hydrolysate; Glu, Glutamine; GA, Gum Arabic.

**P < 0.01.

**Fig 5:**
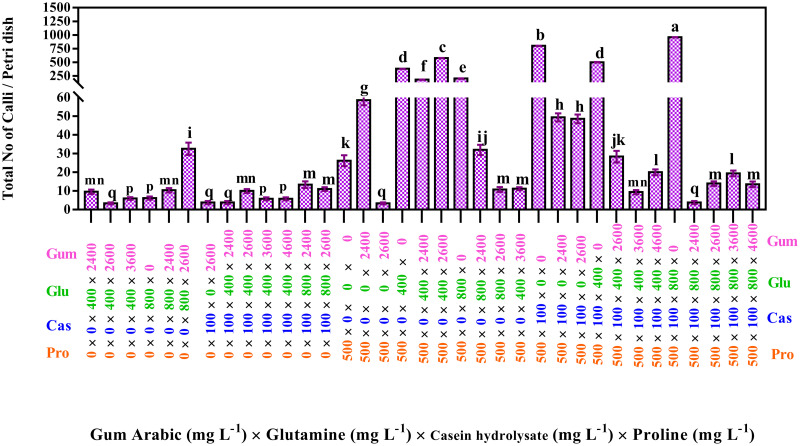
Interaction effects of gum arabic, glutamine, casein hydrolysate and proline on a total number of calli. The studied concentrations include gum arabic (0, 2400, 2600, 3600, 4600, and 5600 mg L^-1^), glutamine (0, 400, and 800 mg L^-1^), casein hydrolysate (0 and 100 mg L^-1^) and proline (0, 100, and 500 mg L^-1^). According to the Kruskal-Wallis test, different letters indicate statistically significant differences.

Results (Fig 5) showed that the highest total number of calli (958.8) was produced by a combination of 800 mg L^-1^ of glutamine, 500 mg L^-1^ of proline, 100 mg L^-1^ of casein hydrolysate, and no gum arabic, followed by a combination of 500 mg L^-1^ of proline, 100 mg L^-1^ of casein hydrolysate (800).

**Fig 6:**
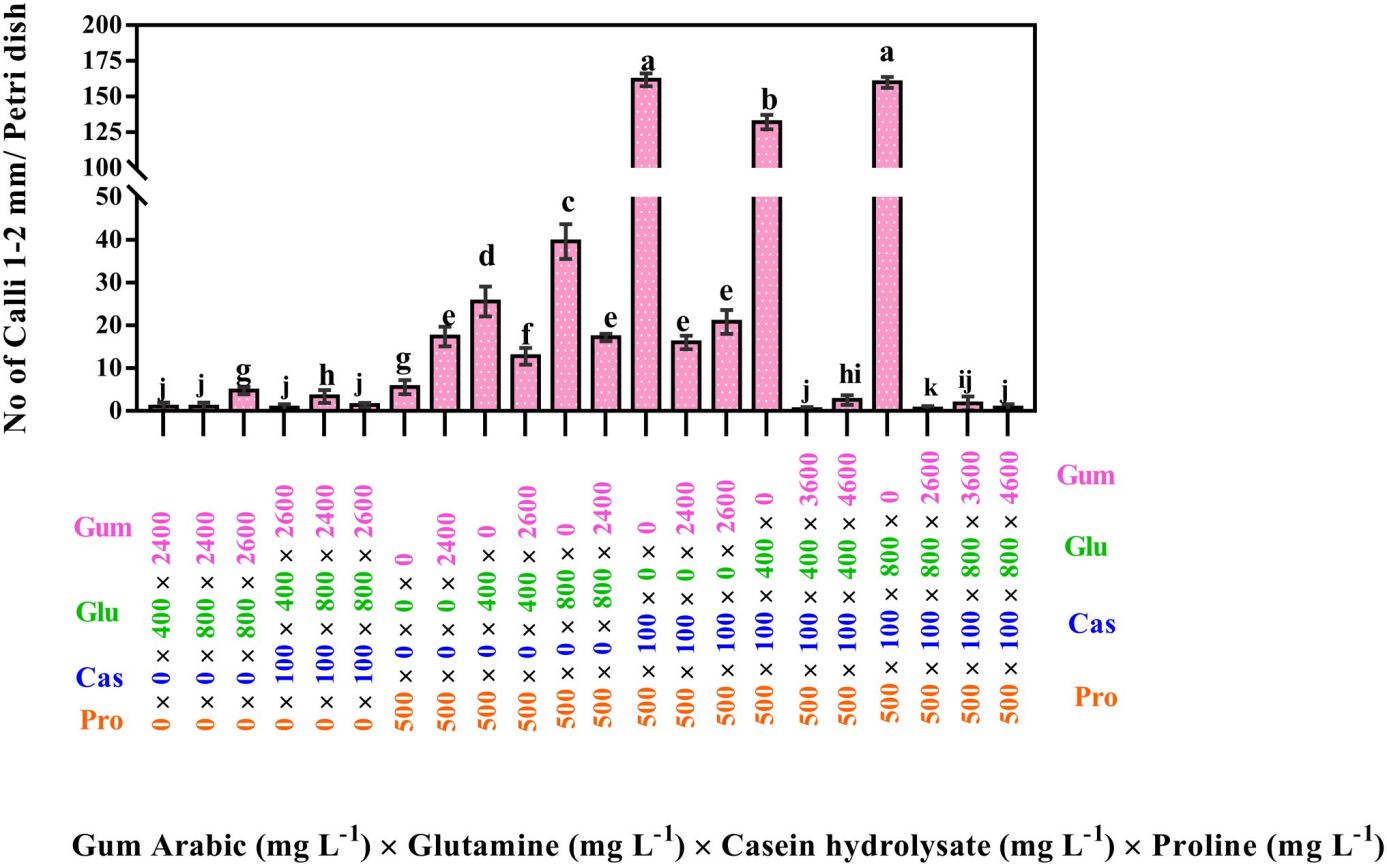
Interaction effects of gum arabic, glutamine, casein hydrolysate and proline on the calli 1–2 mm. The studied concentrations include gum arabic (0, 2400, 2600, 3600, 4600, and 5600 mg L^-1^), glutamine (0, 400, and 800 mg L^-1^), casein hydrolysate (0 and 100 mg L^-1^) and proline (0, 100, and 500 mg L^-1^). According to the Kruskal-Wallis test, different letters indicate statistically significant differences.

Results (Fig 6) also indicated that 500 mg L^-1^ of proline and 100 mg L^-1^ of casein hydrolysate together, as well as the treatment with 500 mg L^-1^ of proline, 100 mg L^-1^ of casein hydrolysate, and 800 mg L^-1^ of glutamine produced the highest number of calli 1–2 mm (161.8 and 160, respectively) followed by a combination of 500 mg L^-1^ of proline, 100 mg L^-1^ of casein hydrolysate, and 400 mg L^-1^ of glutamine, came after these media (132.2).

The interaction effect between 500 mg L^-1^, 100 mg L^-1^ of casein hydrolysate, 800 mg L^-1^ of glutamine, and 0 mg L^-1^ of gum arabic was the best in terms of the total number of calli produced and the number of calli 1–2 mm. By removing glutamine from this treatment, the number of androgenic structures decreased slightly. On the other hand, the response to microspore androgenesis was greatly reduced by adding different concentrations of gum arabic to this treatment.

## 4. Discussion

Even though it has been reported that eggplant anther culture and access to doubled haploid plants are possible [2], many researchers are more interested in isolated microspore culture than anther culture because it has many advantages over anther culture. In eggplant, the microspores move to the direct embryonic pathway after applying combined stress of starvation and heat, but they stop at the globular stage and transform into calli. So far, the efforts have not successfully removed this barrier [22]. In the present study, we investigated the effects of different combinations and concentrations of proline, glutamine, serine, alanine, casein hydrolysate, and gum arabic on eggplant microspore culture.

Cells require nitrogen to make molecules such as amino acids, nucleotides, vitamins, and amino sugars [13]. Most plants usually synthesize the required amino acids for optimal growth; however, the addition of certain amino acids or amino acid mixtures is significant for establishing *in vitro* cultures. Amino acids provide plant cells with a source of nitrogen that is easily assimilated by tissues and cells faster than inorganic nitrogen sources [23]. Amino acids are the building blocks of proteins in living organisms [24]. Also, when energy requirements and glucose levels are high and low, the cells can metabolize amino acids from organic nitrogen sources to gain energy [13]. In most cases, adding organic nitrogen (amino acids) improved non-zygotic embryogenesis [25,26].

Overall, the results of both experiments indicated that a combination of 500 mg L^-1^ of proline, 800 mg L^-1^ of glutamine, 100 mg L^-1^ of serine, and 100 mg L^-1^ of casein hydrolysate produced the highest total number of calli per Petri dish (938 and 958.8, respectively). Based on what we found, 500 mg L^-1^ of proline improved eggplant microspore androgenesis. Androgenic embryogenesis in wheat (*Triticum aestivum* L.) [27], Sorghum (*Sorghum bicolor* L. Moench) [28], and rapeseed (*Brassica napus* L.) [29] has also been shown to be improved by proline. In addition to its role in protein synthesis and the plant cells’ response to environmental stresses, circumstantial evidence suggests that proline may also play a role in development as a metabolite and signal molecule. Some findings are consistent with the role of proline in embryo development [30].

Furthermore, our results also indicated that some of the androgenic structures produced in the medium with 500 mg L^-1^ of proline and without glutamine had a globular shape with a compact appearance. The development of compact and yellow embryonic calli has been observed in sugarcane by adding proline to the induction medium [31]. Proline can also affect plant endogenous hormones as it has promoted the development of early embryonic cell masses in carrots by raising the levels of endogenous hormones, thereby increasing cell mitotic activity [32]. Our study also showed that the higher proline concentration (900 mg L^-1^) did not induce the androgenesis response.

The positive effect of glutamine on the induction and production of microspore-derived calli in eggplant was also observed in our study. Glutamine is involved in synthesizing other amino acids and is used as a nitrogen transporter [33]. It is a key part of the process of nitrogen uptake because it is a mediator in the transport of ammonia to amino acids [13]. Nitrogen metabolism was induced mainly via glutamine and asparagine, serving the former as a substrate to produce the latter [34]. Glutamine is one of the easiest amino acids to get, and many rapidly dividing cells use it as their main energy source under *in vitro* conditions. This amino acid supports the cell’s growth, which needs a lot of energy and synthesizes large amounts of proteins and nucleic acids [13].

According to our results, casein hydrolysate increased the total number of microspore-derived calli. The casein hydrolysate-supplemented media have also been used during the androgenesis process in some plants, including barley (*Hordeum vulgare* L.) [35], European radish [36], and carrot (*Daucus carota* L.) [37]. Casein hydrolysate can act as a nutrient supplement in culture media. It contains mineral elements (such as calcium and phosphate) and, most importantly, a mixture of up to 18 amino acids [38]. The ratio of its amino acids depends on the composition of the protein source from which it is prepared [39].

As the results showed, the microspore-derived calli increased when glutamine, proline, casein hydrolysate, and serine were all present at the same time. Serine is another important amino acid in plants, which is not only a proteinogenic amino acid, but also takes part in catalytic functions of diverse enzymatic reactions in plants. L-Serine participates in the biosynthesis of several biomolecules, including amino acids, phospholipids, and sphingolipids, necessary for cell proliferation [40].

In the second experiment, the highest total number of calli (958.8) was produced by a combination of 800 mg L^-1^ of glutamine, 500 mg L^-1^ of proline, 100 mg L^-1^ of casein hydrolysate, and without gum arabic. Eggplant microspore androgenesis was not improved using the studied concentrations of gum arabic. The effects of gum arabic were significantly influenced by casein hydrolysate and glutamine concentrations. The gum arabic contains a complex polysaccharide consisting of galactose (~40.9%), arabinose (~25.25%), rhamnose (~12%), and glucuronic acid (~15.5%). This gum also contains a small amount of protein (~2.1%) as an integral part of the structure. Its protein contains a mixture of 17 amino acids [41]. When using gum arabic and casein hydrolysate together, it should be noted that both are complex organic compounds, each containing a wide range of different substances. Therefore, microspore androgenesis was affected by the combination of these substances and their concentrations. The results also showed that proline was the most important amino acid in the best treatments, whether the medium has gum arabic or not.

Overall, this study provided insight into the importance of optimizing the concentrations of casein hydrolysate and the studied amino acids to improve eggplant microspore culture.

## 5. Conclusion

In general, this research showed that some amino acids work well to increase the number of microspores-derived calli in eggplant. The calli formed in the medium with 500 mg L^-1^ of proline alone or with serine or casein hydrolysate were globular and compact, especially in the medium with 500 mg L^-1^ of proline, 100 mg L^-1^ of casein hydrolysate, and 100 mg L^-1^ of serine. In addition, the results also showed that the positive effects of amino acids depended on their concentrations and combinations. Therefore, it is necessary to optimize them for each commercial genotype.

## Supporting information

S1 Table Effects of the concentrations of glutamine, alanine, serine, casein hydrolysate, and proline on the eggplant microsporederived calli. Each value is the mean ± SE of five replicates.
(PDF)

S2 Table Effects of the concentrations of proline, casein hydrolysate, glutamine, and gum Arabic on the eggplant microspore-derived calli. Each value is the mean ± SE of five replicates.
(PDF)
