# Supplementary material for: Improving the isolated microspore culture in eggplant (Solanum melongena L.) with amino acid nutrition
Source: PLoS One. 2023 Jun 8;18(6):e0286809. doi: 10.1371/journal.pone.0286809 (PMC10249880; doi:10.1371/journal.pone.0286809)
Supplement: S1 Table — Each value is the mean ± SE of five replicates. (PDF) [file pone.0286809.s001.pdf]

**S1 Table.** Effects of the concentrations of glutamine, alanine, serine, casein hydrolysate, and proline on the eggplant microspore-derived calli. Each value is the mean ± SE of five replicates.

| Treatments (mg $L^{-1}$) | Total No of Calli / Petri dish | No of Calli 1-2 mm/ Petri dish | No of Calli > 2 mm/ Petri dish |
|---|---|---|---|
| Glu (0) × Ala (0) × Ser (0) × CH (0) × Pro (0) | 0 | 0 | 0 |
| Glu (0) × Ala (0) × Ser (0) × CH (0) × Pro (100) | 107.6 ± 11.6 | 27.4 ± 4.9 | 1.2 ± 0.6 |
| Glu (0) × Ala (0) × Ser (0) × CH (0) × Pro (500) | 27 | 0.2 ± 0.09 | 0 |
| Glu (0) × Ala (0) × Ser (0) × CH (0) × Pro (900) | 0 | 0 | 0 |
| Glu (0) × Ala (0) × Ser (0) × CH (100) × Pro (0) | 0 | 0 | 0 |
| Glu (0) × Ala (0) × Ser (0) × CH (100) × Pro (100) | 0 | 0 | 0 |
| Glu (0) × Ala (0) × Ser (0) × CH (100) × Pro (500) | 439.4 ± 46.5 | 124.2 ± 10.3 | 0 |
| Glu (0) × Ala (0) × Ser (0) × CH (100) × Pro (900) | 0 | 0 | 0 |
| Glu (0) × Ala (0) × Ser (100) × CH (0) × Pro (0) | 0 | 0 | 0 |
| Glu (0) × Ala (0) × Ser (100) × CH (0) × Pro (100) | 0 | 0 | 0 |
| Glu (0) × Ala (0) × Ser (100) × CH (0) × Pro (500) | 332.6 ± 21.3 | 87.2 ± 10.8 | 11.2 ± 1.3 |
| Glu (0) × Ala (0) × Ser (100) × CH (0) × Pro (900) | 0 | 0 | 0 |
| Glu (0) × Ala (0) × Ser (100) × CH (100) × Pro (0) | 0 | 0 | 0 |
| Glu (0) × Ala (0) × Ser (100) × CH (100) × Pro (100) | 0 | 0 | 0 |
| Glu (0) × Ala (0) × Ser (100) × CH (100) × Pro (500) | 814.4 ± 23.5 | 152.4 ± 24.7 | 1 ± 0.3 |
| Glu (0) × Ala (0) × Ser (100) × CH (100) × Pro (900) | 0 | 0 | 0 |
| Glu (0) × Ala (100) × Ser (0) × CH (0) × Pro (0) | 0 | 0 | 0 |
| Glu (0) × Ala (100) × Ser (0) × CH (0) × Pro (100) | 0 | 0 | 0 |
| Glu (0) × Ala (100) × Ser (0) × CH (0) × Pro (500) | 7 ± 2.4 | 0.2 ± 0.1 | 0 |
| Glu (0) × Ala (100) × Ser (0) × CH (0) × Pro (900) | 0 | 0 | 0 |
| Glu (0) × Ala (100) × Ser (0) × CH (100) × Pro (0) | 2.6 ± 2 | 0 | 0 |
| Glu (0) × Ala (100) × Ser (0) × CH (100) × Pro (100) | 83 ± 4.7 | 34.8 ± 6.6 | 3.4 ± 1.3 |
| Glu (0) × Ala (100) × Ser (0) × CH (100) × Pro (500) | 146.8 ± 10 | 100.2 ± 15.4 | 1.8 ± 0.8 |
| Glu (0) × Ala (100) × Ser (0) × CH (100) × Pro (900) | 0 | 0 | 0 |
| Glu (0) × Ala (100) × Ser (100) × CH (0) × Pro (0) | 0 | 0 | 0 |

| | | | |
|---|---|---|---|
| Glu (0) × Ala (100) × Ser (100) × CH (0) × Pro (100) | 0 | 0 | 0 |
| Glu (0) × Ala (100) × Ser (100) × CH (0) × Pro (500) | 108.6 ± 18.6 | 48.8 ± 17.4 | 1.6 ± 0.3 |
| Glu (0) × Ala (100) × Ser (100) × CH (0) × Pro (900) | 0 | 0 | 0 |
| Glu (0) × Ala (100) × Ser (100) × CH (100) × Pro (0) | 0 | 0 | 0 |
| Glu (0) × Ala (100) × Ser (100) × CH (100) × Pro (100) | 0 | 0 | 0 |
| Glu (0) × Ala (100) × Ser (100) × CH (100) × Pro (500) | 284.2 ± 16.3 | 55.6 ± 7.3 | 13.8 ± 4.1 |
| Glu (0) × Ala (100) × Ser (100) × CH (100) × Pro (900) | 0 | 0 | 0 |
| Glu (800) × Ala (0) × Ser (0) × CH (0) × Pro (0) | 7.2 ± 2.6 | 2.2 ± 1 | 0.6 ± 0.2 |
| Glu (800) × Ala (0) × Ser (0) × CH (0) × Pro (100) | 53.6 ± 8.1 | 22.4 ± 5.7 | 4.6 ± 1.6 |
| Glu (800) × Ala (0) × Ser (0) × CH (0) × Pro (500) | 221.4 ± 30.4 | 43.8 ± 10.8 | 0.4 ± 0.1 |
| Glu (800) × Ala (0) × Ser (0) × CH (0) × Pro (900) | 0 | 0 | 0 |
| Glu (800) × Ala (0) × Ser (0) × CH (100) × Pro (0) | 120.8 ± 13.7 | 56.4 ± 9.4 | 0 |
| Glu (800) × Ala (0) × Ser (0) × CH (100) × Pro (100) | 91.6 ± 6.2 | 25.8 ± 7.3 | 16 ± 5.5 |
| Glu (800) × Ala (0) × Ser (0) × CH (100) × Pro (500) | 0 | 0 | 0 |
| Glu (800) × Ala (0) × Ser (0) × CH (100) × Pro (900) | 0 | 0 | 0 |
| Glu (800) × Ala (0) × Ser (100) × CH (0) × Pro (0) | 0 | 0 | 0 |
| Glu (800) × Ala (0) × Ser (100) × CH (0) × Pro (100) | 0 | 0 | 0 |
| Glu (800) × Ala (0) × Ser (100) × CH (0) × Pro (500) | 0 | 0 | 0 |
| Glu (800) × Ala (0) × Ser (100) × CH (0) × Pro (900) | 0 | 0 | 0 |
| Glu (800) × Ala (0) × Ser (100) × CH (100) × Pro (0) | 0 | 0 | 0 |
| Glu (800) × Ala (0) × Ser (100) × CH (100) × Pro (100) | 0 | 0 | 0 |
| Glu (800) × Ala (0) × Ser (100) × CH (100) × Pro (500) | 938 ± 44.9 | 200.8 ± 44.1 | 0 |
| Glu (800) × Ala (0) × Ser (100) × CH (100) × Pro (900) | 63.6 ± 4.9 | 30.4 ± 5.02 | 0 |
| Glu (800) × Ala (100) × Ser (0) × CH (0) × Pro (0) | 0 | 0 | 0 |
| Glu (800) × Ala (100) × Ser (0) × CH (0) × Pro (100) | 0 | 0 | 0 |
| Glu (800) × Ala (100) × Ser (0) × CH (0) × Pro (500) | 31.8 ± 5.9 | 16.2 ± 3.9 | 1 ± 0.7 |
| Glu (800) × Ala (100) × Ser (0) × CH (0) × Pro (900) | 29 ± 4.3 | 12.6 ± 1.5 | 0 |
| Glu (800) × Ala (100) × Ser (0) × CH (100) × Pro (0) | 62.6 ± 6.4 | 27.4 ± 6.4 | 0 |
| Glu (800) × Ala (100) × Ser (0) × CH (100) × Pro (100) | 0 | 0 | 0 |
| Glu (800) × Ala (100) × Ser (0) × CH (100) × Pro (500) | 116.6 ± 9.6 | 60.6 ± 7.7 | 5.2 ± 1.3 |

| | | | |
|---|---|---|---|
| Glu (800) × Ala (100) × Ser (0) × CH (100) × Pro (900) | 61 ± 9.2 | 28 ± 4.1 | 0 |
| Glu (800) × Ala (100) × Ser (100) × CH (0) × Pro (0) | 0 | 0 | 0 |
| Glu (800) × Ala (100) × Ser (100) × CH (0) × Pro (100) | 36.4 ± 10.3 | 17.6 ± 4.5 | 7.6 ± 2.3 |
| Glu (800) × Ala (100) × Ser (100) × CH (0) × Pro (500) | 0 | 0 | 0 |
| Glu (800) × Ala (100) × Ser (100) × CH (0) × Pro (900) | 34.8 ± 5.6 | 19.8 ± 3.3 | 0 |
| Glu (800) × Ala (100) × Ser (100) × CH (100) × Pro (0) | 0 | 0 | 0 |
| Glu (800) × Ala (100) × Ser (100) × CH (100) × Pro (100) | 0 | 0 | 0 |
| Glu (800) × Ala (100) × Ser (100) × CH (100) × Pro (500) | 0 | 0 | 0 |
| Glu (800) × Ala (100) × Ser (100) × CH (100) × Pro (900) | 74 ± 6 | 32.6 ± 6.9 | 0 |

Glu, Glutamine; Ala, Alanine; Ser, Serine; CH, Casein hydrolysate; Pro, Proline.
