# Supplementary material for: Improving the isolated microspore culture in eggplant (Solanum melongena L.) with amino acid nutrition
Source: PLoS One. 2023 Jun 8;18(6):e0286809. doi: 10.1371/journal.pone.0286809 (PMC10249880; doi:10.1371/journal.pone.0286809)
Supplement: S2 Table — Each value is the mean ± SE of five replicates. (PDF) [file pone.0286809.s002.pdf]

S2 Table. Effects of the concentrations of proline, casein hydrolysate, glutamine, and gum Arabic on the eggplant microspore-derived calli. Each value is the mean ± SE of five replicates.

**Treatment (mg L^{-1})**	**Total No of Calli / Petri dish**	**No of Calli 1-2 mm/ Petri dish**
Pro (0) × CH (0) × Glu (0) × GA (0)	0	0
Pro (0) × CH (0) × Glu (0) × GA (2400)	0	0
Pro (0) × CH (0) × Glu (0) × GA (2600)	0	0
Pro (0) × CH (0) × Glu (0) × GA (3600)	0	0
Pro (0) × CH (0) × Glu (0) × GA (4600)	0	0
Pro (0) × CH (0) × Glu (0) × GA (5600)	0	0
Pro (0) × CH (0) × Glu (400) × GA (0)	0	0
Pro (0) × CH (0) × Glu (400) × GA (2400)	9.6 ± 1.1	1 ± 1
Pro (0) × CH (0) × Glu (400) × GA (2600)	3.4 ± 0.5	0
Pro (0) × CH (0) × Glu (400) × GA (3600)	6 ± 0.7	0
Pro (0) × CH (0) × Glu (400) × GA (4600)	0	0
Pro (0) × CH (0) × Glu (400) × GA (5600)	0	0
Pro (0) × CH (0) × Glu (800) × GA (0)	6.2 ± 0.8	0
Pro (0) × CH (0) × Glu (800) × GA (2400)	10.4 ± 1.1	1 ± 1
Pro (0) × CH (0) × Glu (800) × GA (2600)	32.6 ± 3.2	4.8 ± 0.8
Pro (0) × CH (0) × Glu (800) × GA (3600)	0	0
Pro (0) × CH (0) × Glu (800) × GA (4600)	0	0
Pro (0) × CH (0) × Glu (800) × GA (5600)	0	0
Pro (0) × CH (100) × Glu (0) × GA (0)	0	0
Pro (0) × CH (100) × Glu (0) × GA (2400)	0	0
Pro (0) × CH (100) × Glu (0) × GA (2600)	3.8 ± 0.8	0
Pro (0) × CH (100) × Glu (0) × GA (3600)	0	0
Pro (0) × CH (100) × Glu (0) × GA (4600)	0	0
Pro (0) × CH (100) × Glu (0) × GA (5600)	0	0
Pro (0) × CH (100) × Glu (400) × GA (0)	0	0

Pro (0) × CH (100) × Glu (400) × GA (2400)	3.8 ± 0.8	0
Pro (0) × CH (100) × Glu (400) × GA (2600)	10	0.8 ± 0.8
Pro (0) × CH (100) × Glu (400) × GA (3600)	5.8 ± 0.8	0
Pro (0) × CH (100) × Glu (400) × GA (4600)	5.8 ± 0.8	0
Pro (0) × CH (100) × Glu (400) × GA (5600)	0	0
Pro (0) × CH (100) × Glu (800) × GA (0)	0	0
Pro (0) × CH (100) × Glu (800) × GA (2400)	13.4 ± 1.8	3.4 ± 1.5
Pro (0) × CH (100) × Glu (800) × GA (2600)	11 ± 1	1.4 ± 0.5
Pro (0) × CH (100) × Glu (800) × GA (3600)	0	0
Pro (0) × CH (100) × Glu (800) × GA (4600)	0	0
Pro (0) × CH (100) × Glu (800) × GA (5600)	0	0
Pro (500) × CH (0) × Glu (0) × GA (0)	26.2 ± 2.9	5.6 ± 1.6
Pro (500) × CH (0) × Glu (0) × GA (2400)	58.6 ± 2.7	17.4 ± 2.3
Pro (500) × CH (0) × Glu (0) × GA (2600)	3.4 ± 0.5	0
Pro (500) × CH (0) × Glu (0) × GA (3600)	0	0
Pro (500) × CH (0) × Glu (0) × GA (4600)	0	0
Pro (500) × CH (0) × Glu (0) × GA (5600)	0	0
Pro (500) × CH (0) × Glu (400) × GA (0)	380.4 ± 2.3	25.6 ± 3.5
Pro (500) × CH (0) × Glu (400) × GA (2400)	181.4 ± 2.7	0
Pro (500) × CH (0) × Glu (400) × GA (2600)	576 ± 3.9	12.8 ± 1.9
Pro (500) × CH (0) × Glu (400) × GA (3600)	0	0
Pro (500) × CH (0) × Glu (400) × GA (4600)	0	0
Pro (500) × CH (0) × Glu (400) × GA (5600)	0	0
Pro (500) × CH (0) × Glu (800) × GA (0)	201.6 ± 5.3	39.6 ± 4
Pro (500) × CH (0) × Glu (800) × GA (2400)	32 ± 2.7	17.2 ± 0.8
Pro (500) × CH (0) × Glu (800) × GA (2600)	10.8 ± 1.3	0
Pro (500) × CH (0) × Glu (800) × GA (3600)	11.2 ± 0.8	0
Pro (500) × CH (0) × Glu (800) × GA (4600)	0	0
Pro (500) × CH (0) × Glu (800) × GA (5600)	0	0
Pro (500) × CH (100) × Glu (0) × GA (0)	800 ± 3.4	161.8 ± 4.5

Pro (500) × CH (100) × Glu (0) × GA (2400)	49.4 ± 2.1	16 ± 1.5
Pro (500) × CH (100) × Glu (0) × GA (2600)	48.6 ± 2.3	20.8 ± 2.7
Pro (500) × CH (100) × Glu (0) × GA (3600)	0	0
Pro (500) × CH (100) × Glu (0) × GA (4600)	0	0
Pro (500) × CH (100) × Glu (0) × GA (5600)	0	0
Pro (500) × CH (100) × Glu (400) × GA (0)	500.2 ± 4.4	132.2 ± 5.1
Pro (500) × CH (100) × Glu (400) × GA (2400)	0	0
Pro (500) × CH (100) × Glu (400) × GA (2600)	28.4 ± 3.1	0
Pro (500) × CH (100) × Glu (400) × GA (3600)	9.4 ± 1.1	0.4 ± 0.5
Pro (500) × CH (100) × Glu (400) × GA (4600)	20 ± 1.5	2.6 ± 1.1
Pro (500) × CH (100) × Glu (400) × GA (5600)	0	0
Pro (500) × CH (100) × Glu (800) × GA (0)	958.8 ± 2.5	160 ± 3.8
Pro (500) × CH (100) × Glu (800) × GA (2400)	3.8 ± 0.8	0
Pro (500) × CH (100) × Glu (800) × GA (2600)	14 ± 1.2	0.6 ± 0.5
Pro (500) × CH (100) × Glu (800) × GA (3600)	19.4 ± 1.5	1.8 ± 1.6
Pro (500) × CH (100) × Glu (800) × GA (4600)	13.6 ± 1.5	0.8 ± 0.8
Pro (500) × CH (100) × Glu (800) × GA (5600)	0	0

Pro, Proline; Ch, Casein hydrolysate; Glu, Glutamine; GA, Gum Arabic.
